# X-ray absorption spectroscopy and X-ray diffraction data for molybdenum minerals and compounds

**DOI:** 10.1016/j.dib.2022.108576

**Published:** 2022-09-13

**Authors:** Valerie A. Schoepfer, Matthew B.J. Lindsay

**Affiliations:** Department of Geological Sciences, University of Saskatchewan, Saskatoon, SK S7N 5E2, Canada

**Keywords:** extended X-ray absorption fine structure, X-ray absorption near edge structure, kamiokite, sidwillite, molybdite, bamfordite, lindgrenite, ferrimolybdite, EXAFS, extended X-ray absorption fine structure, XANES, X-ray absorption near edge structure

## Abstract

We report Mo K- and L_III_-edge X-ray absorption spectroscopy (XAS) and X-ray diffraction (XRD) data collected for 15 molybdenum minerals and compounds sourced from museum collections, mineral dealers, and chemical suppliers. The samples were finely ground and analyzed at the Canadian Light Source synchrotron (Saskatoon, Canada). The L_III_-edge XAS data were collected in fluorescence and total electron yield mode, while the K-edge XAS data were collected in transmission and fluorescence modes. Molybdenum L_III_-edge spectra cover the X-ray absorption near edge structure (XANES) region and Mo K-edge spectra cover the extended X-ray absorption fine structure (EXAFS) region. Tabulated XAS data are provided to support analysis of XAS data obtained for geological or environmental research. Furthermore, Mo K-edge EXAFS and L_III_-edge XANES spectra, the k^3^ weighted oscillatory χ(k) functions, and the Fourier-transforms in χ(R) of these K-edge data are presented graphically. Corresponding XRD data were collected as two-dimensional images against an area detector and integrated to form line scans. The data were collected at a wavelength of 0.68866 Å (18 keV) and is tabulated and presented graphically over a 0-40 °2Θ range. This dataset is intended to be used as reference material for a variety of rare and common Mo phases.

**Specifications Table**

**Table:** 

Subject	Environmental Chemistry
Specific subject area	X-ray absorption spectroscopy and X-ray diffraction of common and rare molybdenum minerals and compounds
Type of data	Table Figure
How the data were acquired	All data was collected at the Canadian Light Source synchrotron (Saskatoon, Canada). Molybdenum L_III_-edge XANES data were collected at the SXRMB bending magnet beamline under high vacuum, Mo K-edge EXAFS were collected at the BioXAS-Main wiggler beamline under liquid nitrogen, and X-ray diffraction data were collected at the CMCF beamline. The XAS data were processed using the Demeter XAS data processing package [1], while XRD data were processed using GSAS-II crystallography data analysis software [2].
Data format	Raw Processed
Description of data collection	Common and rare Mo compounds and minerals were ground and thinly spread on carbon coated or Kapton tape. Mo L_III_-edge XANES were collected under high vacuum and Mo K-edge EXAFS spectra were collected under liquid nitrogen at the Canadian Light Source synchrotron in total electron yield, transmission, or fluorescence mode. Synchrotron-based XRD spectra were collected on capillary-packed material.
Data source location	Molybdenite – University of Saskatchewan Mineral Collection, Saskatoon, Saskatchewan, Canada. Sample 2961 K1 collected from Easton, PA, USA (40.69, -75.22) Wulfenite – Collected from La Morita Mine, Chihuahua, Mexico (30.90, -107.60) Powellite – Collected from Pune District, Maharashtra, India (18.52, 73.86) Bamfordite – Museums Victoria, Melbourne, Victoria, Australia. Sample M44484 collected from Bamford Hill W-Mo-Bi deposit, Grandon's Shoot, Norton's lease, Queensland, Australia (-17.31, 144.92). Kamiokite – The University of Arizona Mineral Museum, Tucson, Arizona. Sample 19002 collected from 5^th^ May Adit, Vrchoslav Mine, Krupka, Krušné Hory Mountains, Ústechý, Czech Republic (50.69, 13.83). Ferrimolybdite – Canadian Museum of Nature, Ottawa, Ontario, Canada. Sample CMNMC 40358 collected from Mt. Quyon, Pontian Co., Quebec, Canada (45.51, -76.23). Lindgrenite – Canadian Museum of Nature, Ottawa, Ontario, Canada. Sample CMNMC 57163 collected from Magma Mine, #3 Pit, Arizona, USA 33.3, -111.09). Molybdite – Royal Scottish Museum Department of Geology, Edinburgh, Scotland. Sample G.1884.20.82 collected from Ross, Ontario, Canada (45.67, -76.82). Sidwillite – Royal Scottish Museum Department of Geology, Edinburgh, Scotland. Sample G.1985.3.7 (Dana No. 4.5.6.1) collected from Lake Como, Colorado, USA (37.92, -107.62). Ilsemannite – Royal Ontario Museum, Toronto, Ontario, Canada. Sample ROMESM 3255.1 collected from Himmelsfurst Mine, Mittelsachsen, Saxony, Germany (50.76, 13.30). Mo foil – Standard Mo(0) XAS reference foil purchased from EXAFS Materials (exafsmaterials.com) Mo coprecipitated ferrihydrite – Co-precipitated Na_2_MoO_4_ and ferrihydrite synthesized via rapid titration [3].
	Sodium molybdate – CAS: 7631-95-0, Sigma-Aldrich, >98% Mo(VI) oxide – CAS: 1313-27-5, Alfa-Aesar, 99.9995% Mo(IV) oxide – CAS: 18868-43-4, Sigma-Aldrich, 99%
Data accessibility	Repository name: Federated Research Data Repository Direct URL to data: doi.org/10.20383/103.0600

## Value of the Data

• Extensive molybdenum sequestration is thought to occur in mining locations and in the deep oceans, however, Mo identification and speciation in these situations is limited due to the lack of reference materials.
• Environmental geochemists, paleoclimatologists, and geologists interested in Mo speciation will benefit from these data.
• This dataset is intended to be used as reference material in the identification and characterization of Mo minerals from future studies.

## 1 Data Description

Table 1. A list of details for the reference materials and minerals prepared in this dataset. The table includes the molecular formula, the Mo oxidation state, the space group, crystal system, class, Nickel-Strunz group, IMA-CNMNC symbol [4], and some notes. A reference to crystallographic information files are also listed.

**Table 1:** Individual reference materials and minerals prepared in this dataset.

Reference name	Formula	Mo oxidation state	Space group	Crystal system	Class	Nickel-Strunz group	IMA-CNMNC symbol	Notes
bamfordite^1^	FeMo_2_O_6_(OH)_3_ · H_2_O	VI	P1	Triclinic	Pinacoidal	oxide	Bfd	
Ferrimolybdite	Fe_2_(MoO_4_)_3_ · nH_2_O	VI	Pmmn	Orthorhombic	Dipyramidal	sulfate	Fmyb	
ilsemannite	Mo_3_O_8_	VI		Amorphous		oxide	Ils	amorphous, questionable mineral status
kamiokite^2^	Fe₂Mo₃O₈	IV	P 6_3_mc	Hexagonal	Dihexagonal pyramidal	oxide	Kmk	
lindgrenite^3^	Cu_3_(MoO_4_)_2_(OH)_2_	VI	P2_1_/m	Monoclinic	Prismatic	sulfate	Lgr	
Mo foil	Mo	0				element		commercial
Mo coprecip ferrihydrite	MoO4-Fe(OH)3	VI						molybdate coprecipitated into ferrihydrite
molybdenite^4^	MoS_2_	IV	P6_3_/mmc	Hexagonal	6/mmm	sulfide	Mol	semiconductor, poor fluorescence
molybdenum dioxide	MoO_2_	IV						commercial
molybdenum trioxide	MoO_3_	VI						commercial
molybdite^5^	MoO_3_	VI	Pbnm	Orthorhombic	Dipyramidal	oxide	Myb	limited sample
powellite^6^	CaMoO_4_	VI	I 4_1_/a	Tetragonal	Dipyramidal	sulfate	Pwl	
sidwillite^7^	MoO_3_ · 2H_2_O	VI	P 2_1_/n	Monoclinic	Prismatic	oxide	Sdw	limited sample
sodium molybdate	Na_2_MoO_4_	VI						commercial
wulfenite^8^	PbMoO_4_	VI	I 4_1_/a	Tetragonal	Dipyramidal	sulfate	Wul	

1 Birch W. D., Pring A., Mcbriar E. M., Gatehouse B. M. and Mccammon C. A. (1998) Bamfordite, Fe3+Mo2O6(OH)3·H2O, a new hydrated iron molybdenum oxyhydroxide from Queensland, Australia: Description and crystal chemistry. American Mineralogist 83, 172–177.

2 Kanazawa Y. and Sasaki A. (1986) Structure of kamiokite. Acta Crystallogr C Cryst Struct Commun 42, 9–11.

3 Calvert L. D. and Barnes W. H. (1957) The structure of lindgrenite. The Canadian Mineralogist 6, 21.

4 Schönfeld B., Huang J. J. and Moss S. C. (1983) Anisotropic mean-square displacements (MSD) in single-crystals of 2 H - and 3 R -MoS 2. Acta Crystallogr B Struct Sci 39, 404–407.

5 Wooster N. (1931) The crystal structure of Molybdenum Trioxide, MoO3. Zeitschrift Für Kristallographie - Crystalline Materials 80, 1–6.

6 Hazen R. M., Finger L. W. and Mariathasan J. W. E. (1985) High-pressure crystal chemistry of scheelite-type tungstates and molybdates. Journal of Physics and Chemistry of Solids 46, 253–263.

7 Krebs B. (1972) Die Kristallstruktur von MoO 3 .2H 2 O. Acta Crystallogr B Struct Sci 28, 2222–2231.

8 Leciejewicz J. (1965) A neutron crystallographic investigation of lead molybdenum oxide, PbM 0 O 4. Zeitschrift für Kristallographie - Crystalline Materials 121, 158–164.

**Fig. 1:**
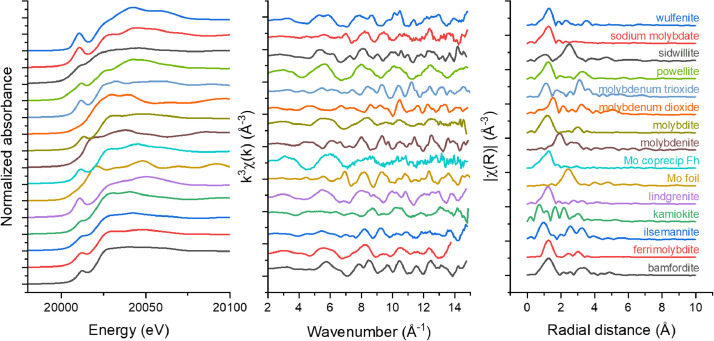
The Mo K-edge XAS spectra displayed in a) energy space for the XANES region, b) k space for the EXAFS region, and c) R-space for the EXAFS region.

Fig. 1. a) The Mo K-edge XAS spectra collected for the XANES region of the 15 reference materials, obtained over the energy range –20 eV to +100 eV around the Mo K-edge (20,000 eV) and collected at the BioXAS beamline at the CLS. All data is reported as edge-step normalized absorbance and was collected in fluorescence mode with the exception of the Mo foil and wulfenite which were collected in transmission mode. b) The k^3^ weighted k-space equivalent of the EXAFS region of each of the references, reported from 2-15 Å^−1^. c) The corresponding R-space for each reference,m where data is shown from 0-10 Å. The Fourier Transform was conducted over the k-space of 3-11 Å^−1^.

**Fig. 2:**
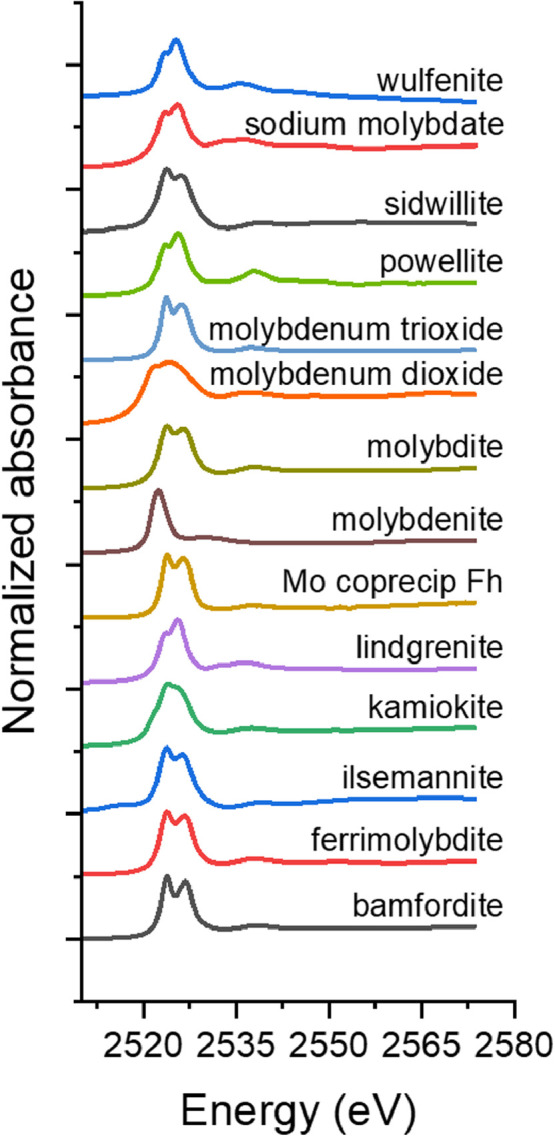
The Mo L_III_-edge XANES spectra for reference materials..

Fig. 2. The Mo L_III_-edge XAS spectra was collected for the XANES region, where data was collected from -30 eV to +55 eV around the Mo L_III_-edge (2525 eV) at the SXRMB beamline at the CLS. All files were collected in fluorescence mode with the exception of molybdenite, which was collected in TEY. All data is reported as edge-step normalized absorbance.

**Fig. 3:**
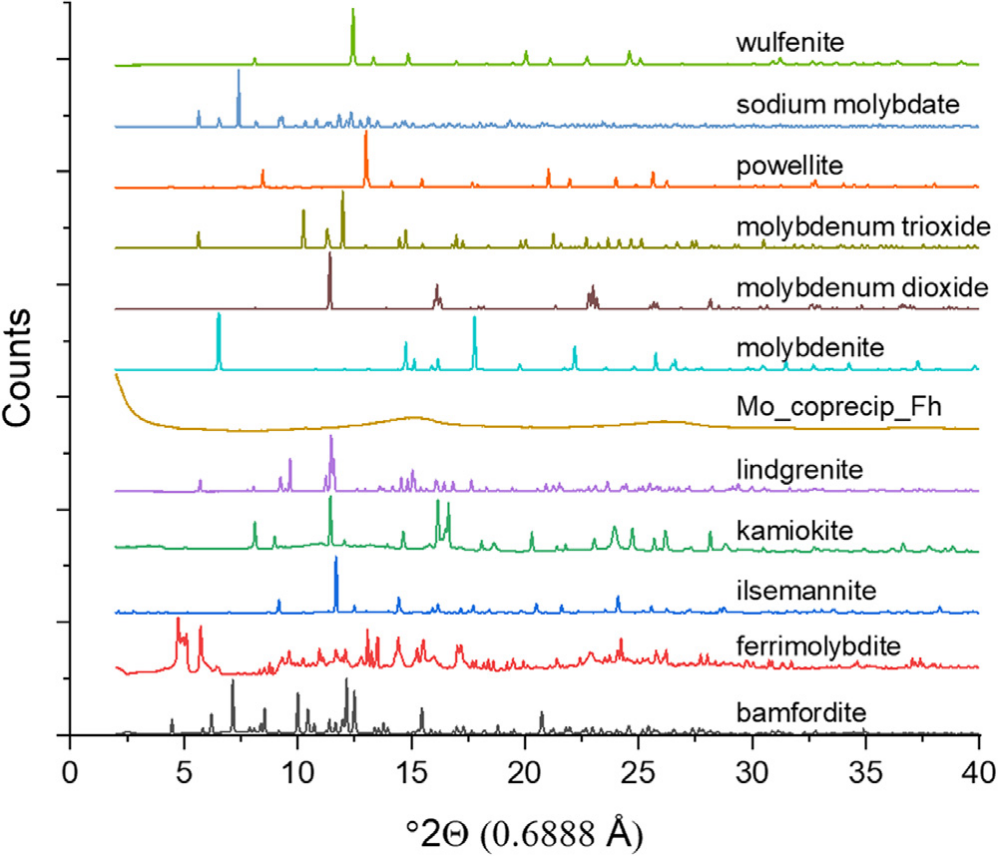
X-ray diffraction data for reference materials collected at 0.6888 Å.

Fig. 3. X-ray diffraction data for each reference material with the exception of the Mo X-ray absorption foil, molybdite and sidwillite, which were not collected because of limited material. Data was collected at a wavelength of 0.6888 Å on an 18 keV beam at the CMCF beamline at the CLS.

Dataset 1. Mo K-edge energy calibrated Fluorescence XAS data collected from -200 eV to 15 k around the Mo K-edge (20,000 eV). This folder contains an Athena project file containing all references collected in fluorescence mode when possible. A subfolder lists each individual spectra as an .xmu file. These individual files are energy calibrated but not normalized to preserve the edge jump. Incident energy, measured in the first ionization chamber (I_0_) and often noted as ‘Energy Feedback’, is listed in the first column, the absorption data of the desired reference in the second, and a Mo(0) reference foil is presented in the third column and was collected between chambers I_1_ and I_2_ for each scan to allow for alignment between scans.

Dataset 2. Mo K-edge energy calibrated Transmission XAS data collected from -200 eV to 15 k around the Mo K-edge (20,000 eV). This folder contains an Athena project file containing all references collected in transmission mode when possible. A subfolder lists each individual spectra as an .xmu file. These individual files are energy calibrated but not normalized to preserve the edge jump. Incident energy, measured in the first ionization chamber (I_0_) and often noted as ‘Energy Feedback’, is listed in the first column, the transmission data of the desired reference in the second, and a Mo(0) reference foil is presented in the third column and was collected between chambers I_1_ and I_2_ for each scan to allow for alignment between scans.

Dataset 3. Mo L_III_-edge energy calibrated Fluorescence XANES data was collected from -30 eV to +55 eV around the Mo L_III_-edge (2525 eV). This folder contains an Athena project file containing all references collected in fluorescence mode when possible. A subfolder lists each individual spectra as an .xmu file. These individual files are energy calibrated but not normalized to preserve the edge jump. Incident energy, measured in the first ionization chamber (I_0_) and often noted as ‘Energy Feedback’, is listed in the first column and the absorption data of the desired reference in the second. Subsequent columns record the pre- and post-edge and the derivative of the spectra. Mo(0) foil was not collected.

Dataset 4. Mo L_III_-edge energy calibrated Total Electron Yield XANES data was collected from -30 eV to +55 eV around the Mo L_III_-edge (2525 eV). This folder contains an Athena project file containing all references collected in Total Electron Yield mode when possible. A subfolder lists each individual spectra as an .xmu file. These individual files are energy calibrated but not normalized to preserve the edge jump. Incident energy, measured in the first ionization chamber (I_0_) and often noted as ‘Energy Feedback’, is listed in the first column and the TEY data of the desired reference in the second. Subsequent columns record the pre- and post-edge and the derivative of the spectra. Mo(0) foil was not collected.

Dataset 5. Mo compound XRD was collected at 18 keV (λ = 0.6888 Å). This folder contains .asc files of the XRD patterns of each available reference from 2-40 °2θ. Sidwillite, molybdite, and the Mo foil were not collected due to limited material. Select files are not confirmed with a published crystallographic information file (CIF), and are presented as is. The first column represents the °2θ and the second represents the intensity, while the third column is the error term.

## 2 Experimental Design, Materials and Methods

**Reference material preparation** – Following a visual assessment for uniformity, samples were finely ground using an agate mortar and pestle.

**L_III_-edge XANES analysis** – About 20 μg of finely ground reference materials were pressed onto a conductive Cu plate using double sided carbon tape as an adhesive. The total area of each sample was approximately 1-2 cm^2^ and several materials were affixed to one copper plate, taking care to avoid contamination during preparation and transport.

X-ray absorption spectroscopy (XAS) data was collected at the Canadian Light Source (CLS) in Saskatoon, Saskatchewan, Canada. The storage ring was operated in continuous top-up mode to a current of 220 mA. We collected XAS data over the X-ray absorption near-edge structure (XANES) region of the Mo L_III_-edge. Total electron yield (TEY) as well as fluorescence yield data for the Mo L_III_ edge were obtained at the SXRMB bending magnet beamline (06B1-1) under a vacuum of ∼5 × 10^−8^ Torr. The sample was mounted at 45 degrees to the incoming beam and another 45 degrees to the 7-element fluorescence detector. This beamline has an energy resolution of 1 × 10^−4^ eV using a dual Si(111) crystal monochromator, a flux > 1 × 10^11^ photons/sec, and a spot size approximately 1 × 4 mm, focused using a toroidal mirror. Scans ranged from -30 eV to -5 eV at 2 eV steps in the pre-edge region, from -5 eV to +25 eV at 0.15 eV steps in the near-edge region, and from +25 eV to +55 eV at 0.75 eV steps in the post-edge region, relative to the Mo L_III_-edge (2525 eV). Two scans were collected for each reference material to ensure replicability.

**K-edge XANES and EXAFS analysis** – Approximately 20 μg of finely ground reference material was adhered as a thin layer to polyimide (Kapton) tape, folded onto itself, and enclosed in another layer of polyimide tape, to create two layers of each reference material. This method was designed to reduce pinhole effects and self-absorption, while allowing sufficient signal transmission. Several reference materials were not abundant enough to achieve two layers, and therefore one thin layer was used.

Molybdenum K-edge X-ray absorption spectroscopy, including XANES and extended X-ray absorption fine structure (EXAFS) was performed at the BioXAS-Main wiggler beamline (07ID-2) at the CLS. This beamline has an energy resolution of < 1 × 10^−4^ eV with a flux > 1 × 10^12^ photons/second and a spot size approximately 3 × 0.5 mm, focused using Rh-coated toroidal mirrors while a Si(220) double crystal monochromator selects the energy. The setup includes three ionization chambers and two Ge-32 element fluorescence detectors, along with a liquid nitrogen cryostat, Soller slits, and a Zr6 fluorescence filter to enhance the signal to noise ratio. Spectra were collected in fluorescence mode, and additional transmission spectra were collected when enough sample was present. Fluorescence and transmission scans ranged from -200 eV below the theoretical Mo K edge (20,000 eV) to k=15 Å^−1^ at 5 eV steps in the pre edge region (i.e., 19800–19900 eV), 0.5 eV steps in the XANES region (i.e., 19900–21000 eV), and 0.05 k in the EXAFS region (i.e., 21000 eV to 15 Å^−1^). At least two scans per samples ensured replicability, while a Mo reference foil was collected downstream from the sample at each scan for energy calibration and alignment.

**XAS Data preparation and treatment** – Data analysis was conducted using the Athena program in the Demeter software package [1]. Raw scans were corrected for the total flux in I_0_ and imported into Athena as μ(E) as either fluorescence or transmission data for the K-edge or imported as fluorescence or total electron yield data for the L_III_-edge. Each scan was quality checked and individually deglitched when necessary. Replicate scans were aligned to each other and merged. When one file remained for each reference material, the centroid of the pre-edge peak in the sodium molybdate scan was calibrated to 20010.5 eV for the K-edge and 2523.5 eV for the L_III_-edge [5], and all other files from the same beamtime were aligned to this file using an energy shift or the associated Mo(0) reference foils. This results in the K-edge Mo(0) foil having an approximate E_0_ of 20016.5 eV. An E_0_ value for each individual spectra was selected as the highest point in the main peak of the first derivative. Due to a strong pre-edge peak associated with Mo(VI), this selection was not always on the first peak in the first derivative, but the peak associated with the main rising edge in μ(E). The pre-edge range was determined at the L_III_ and K-edges visually, by selecting as large a range as possible while maintaining a relatively flat line. The post-edge, edge-step normalization range used the automatically selected lower bound, as this bound is typically accurate, while manually extending the upper bound of this range as far as the data allowed, and these selections were modified if necessary after viewing the normalized spectra. The normalization step was one arbitrary absorbance unit. Data are reported as the I_0_ corrected, energy calibrated, non-normalized μ(E) data, with this data retaining the edge-step associated with each reference material, as concentrations and signal intensity varied per reference material and by analysis mode. However, data normalized to the edge step are presented visually.

When preparing to plot the χ(k) and χ(R) space for the K-edge spectra, the R background was selected to be between 0.7 and 1.0 with a k-weight of 2 and these values remained constant throughout scans of the same beamtime. This R background value was chosen to minimize the peak in the background function that appeared at approximately k = 1, while also not allowing the background function to invert at k < 1. However, as the forward Fourier transform k-range was set from 3.0-11.0 and data/background below the k-range of 3.0 is ignored, the R background function at these low k values was not highly important. The spline range for the R background function extended throughout the entirety of the dataset from 0-15 k, and corresponded to a spline range in E from 0-857 eV. There was no spline clamp in the low energy range, while a strong spline clamp was used in the high energy range to constrain the background function. The forward Fourier transform parameters used a k-range from 3.0-11.0 with a dk of 3 in a Hanning window, while the backwards Fourier transform was from 1-3 with a dR of 0.0 in a Hanning window.

**X-ray diffraction** – Synchrotron based powder x-ray diffraction (XRD) for all materials was conducted on the CMCF-BM beamline (08-B1-1) in both manual mode and remote access mode using the Stanford Automated Mounting system. Finely ground powder samples were loaded into 0.5 mm inner diameter polyimide (Kapton) capillaries around 2.2 cm in length. These capillaries were sealed on both ends with ethyl cyanoacrylate adhesive, loaded into magnetic Unipuck stubs, placed on a SSRL cassette, and transported to the CLS where it was mounted for access manually or by a robotic arm onto the goniometer. Capillaries were rotated during the 10-60 second exposure to the 18 keV (λ = 0.6888 Å) beam at room temperature, resulting in a 2Θ range of 2-40°. The detector was at a distance of 350 mm and used an aperture of 200 μm. Three to six two-dimensional scans were collected using a high resolution Pilatus3 S 6M X-ray detector. The flux at this beamline is approximately 1.5 × 10^11^ photons/second with an energy resolution of ∼1.5 × 10^−4^ eV.

The two-dimensional, concentric images were processed using the program GSAS-II [2], where instrument parameters, including the exact incident wavelength, were calibrated using a LaB_6_ calibrant. Following calibration, sample files were imported into GSAS-II and background subtracted using an empty capillary of the same diameter exposed for the same duration as the associated sample. Scans were integrated to convert the two-dimensional image to a line scan, and the line scans binned using equal weighting for each. The data was saved as a powder Topas XYE file, which was manually converted to an .asc file. Data is plotted as normalized from 0-1.

## Ethics Statement

The authors declare that the manuscript adheres to ethics in publishing standards.

## CRediT Author Statement

**Valerie A. Schoepfer:** Formal analysis, Validation, Investigation, Data curation, Writing – original draft, Visualization; **Matthew B.J. Lindsay:** Resources, Validation, Writing – review & editing, Supervision, Funding acquisition.

## Declaration of Competing Interest

The authors declare that they have no known competing financial interests or personal relationships that could have appeared to influence the work reported in this paper.

## Data Availability

X -ray absorption spectroscopy and X-ray diffraction data for molybdenum minerals and compounds: Datasets and Supplementary Materials (Original data) (Federated Research Data Repository).
